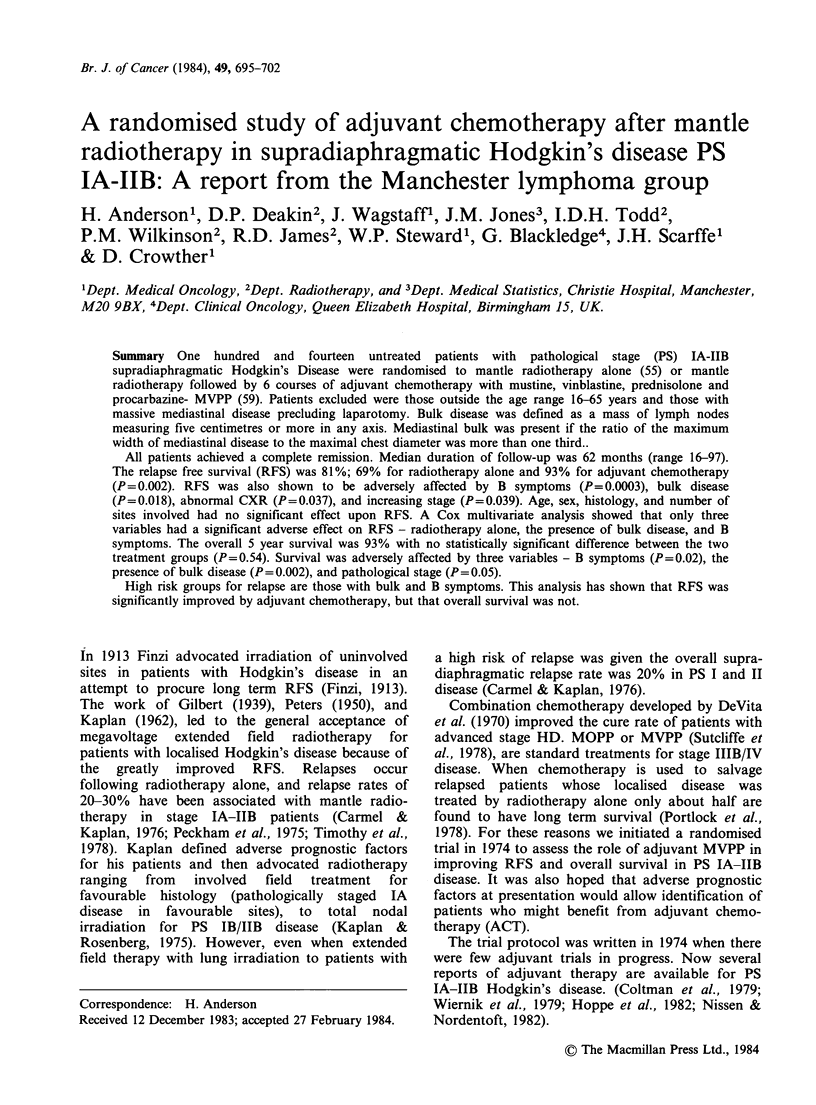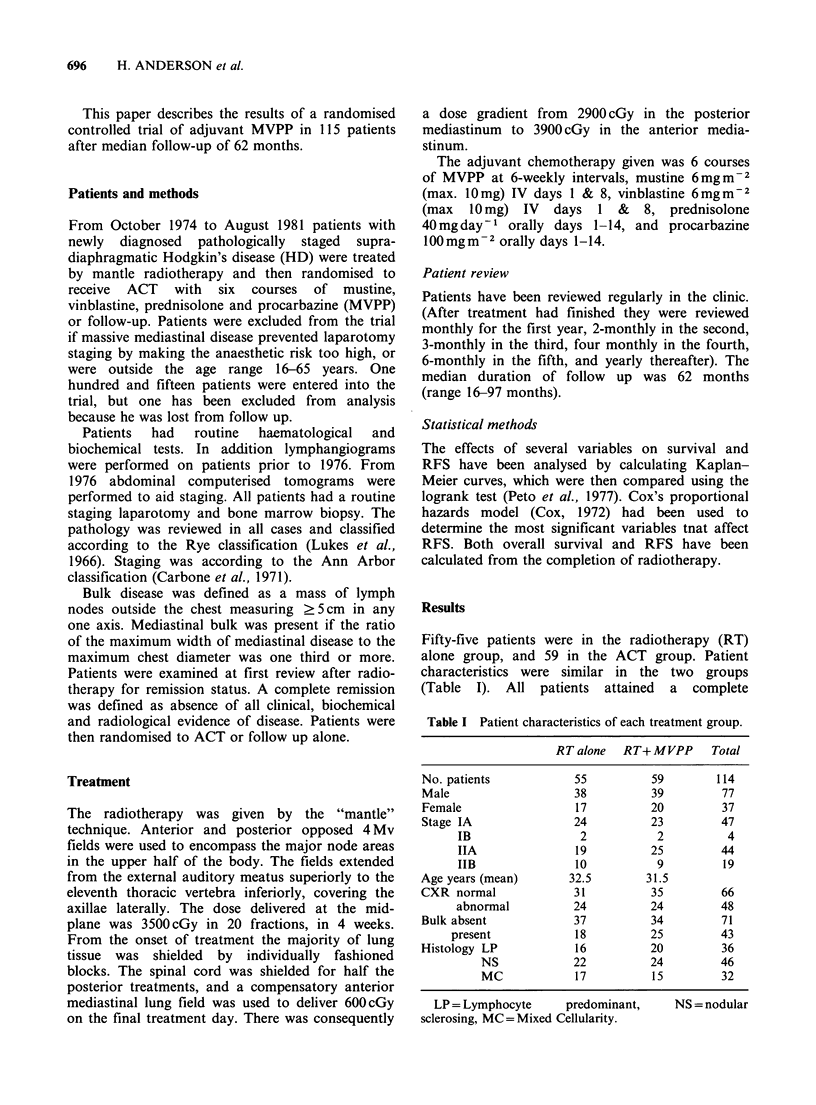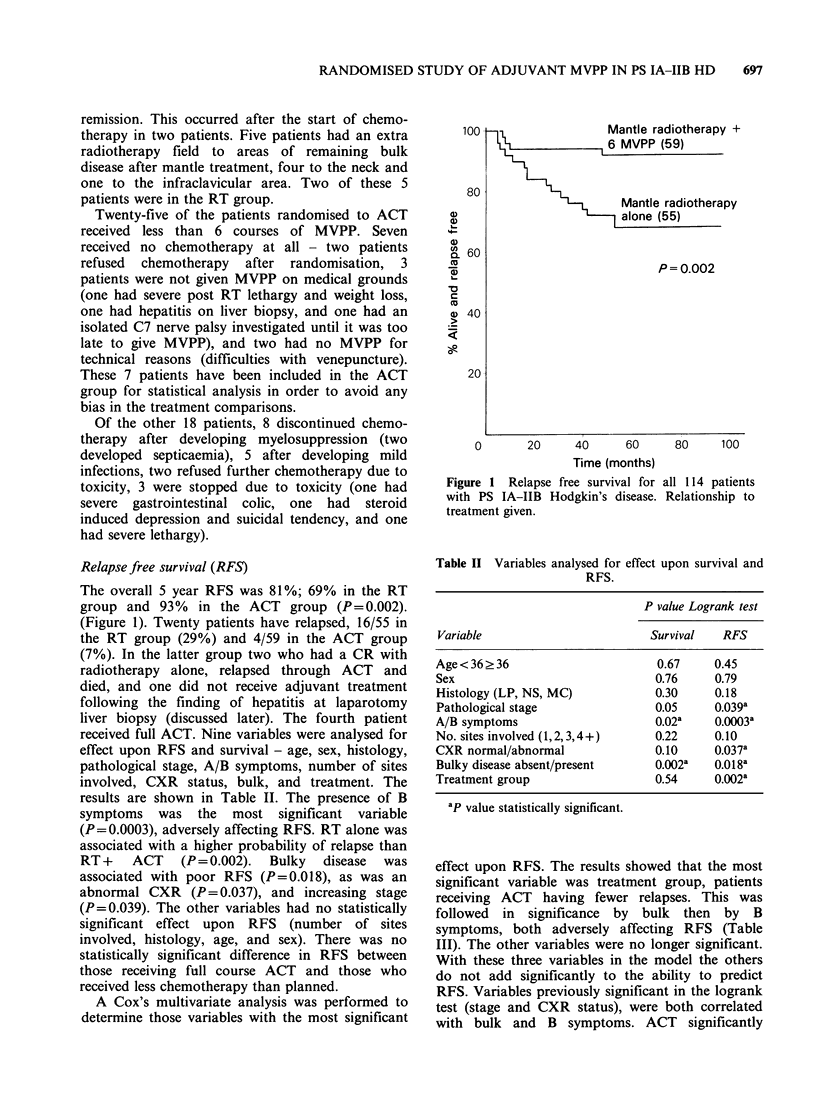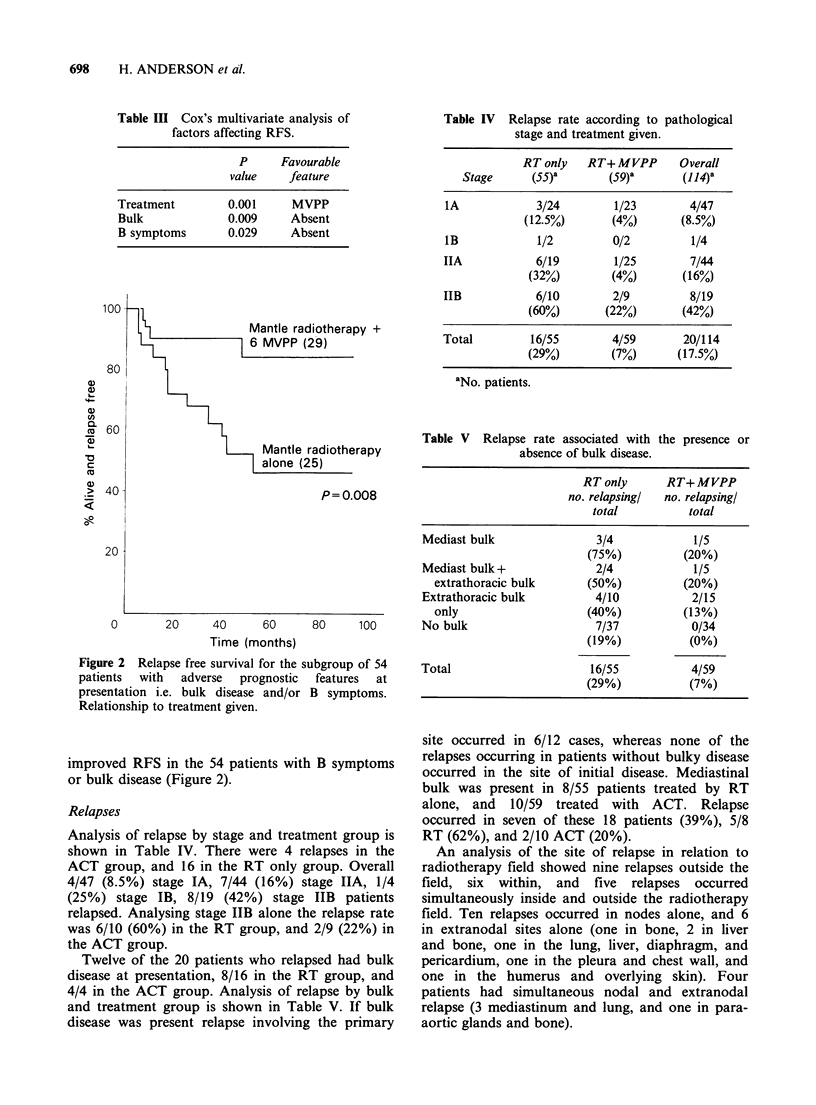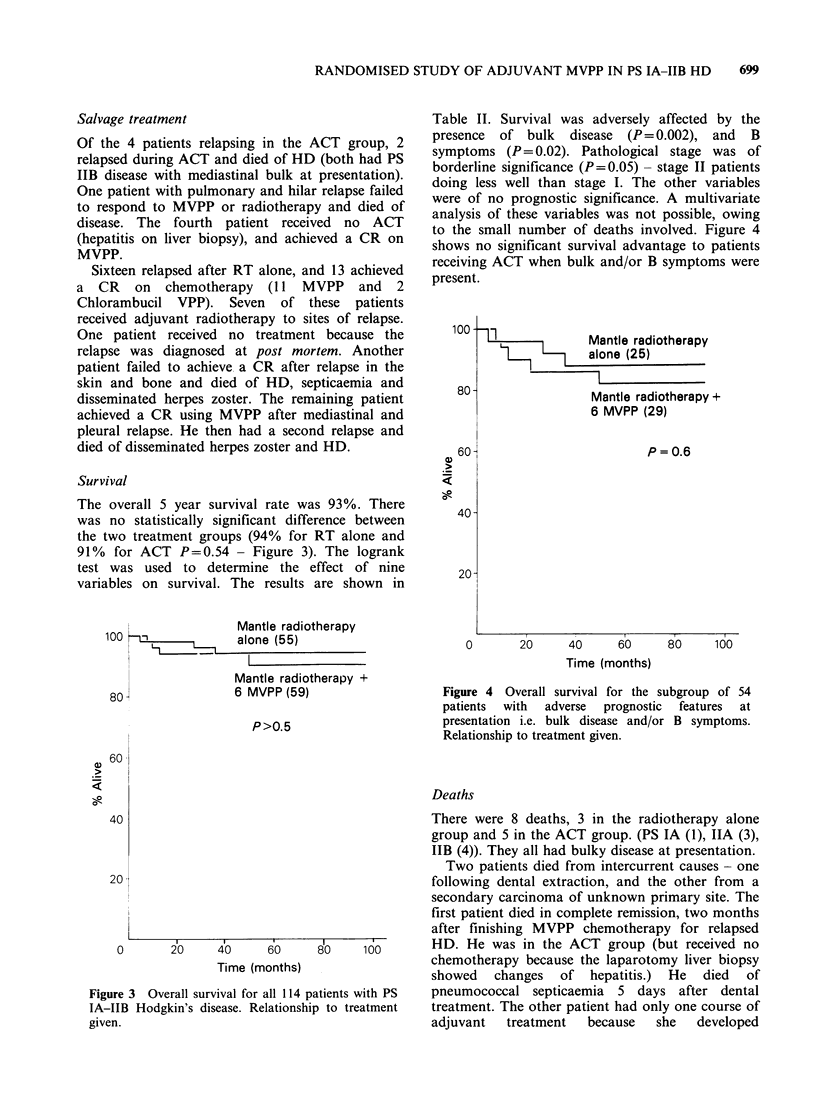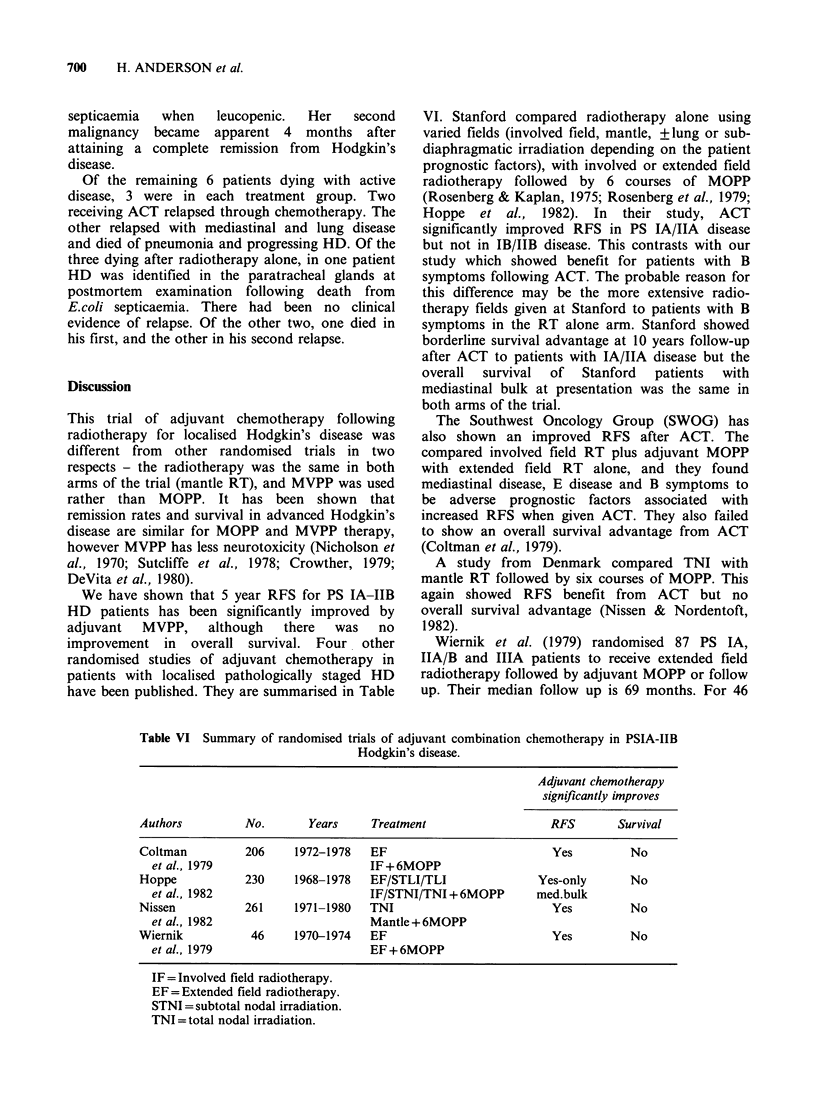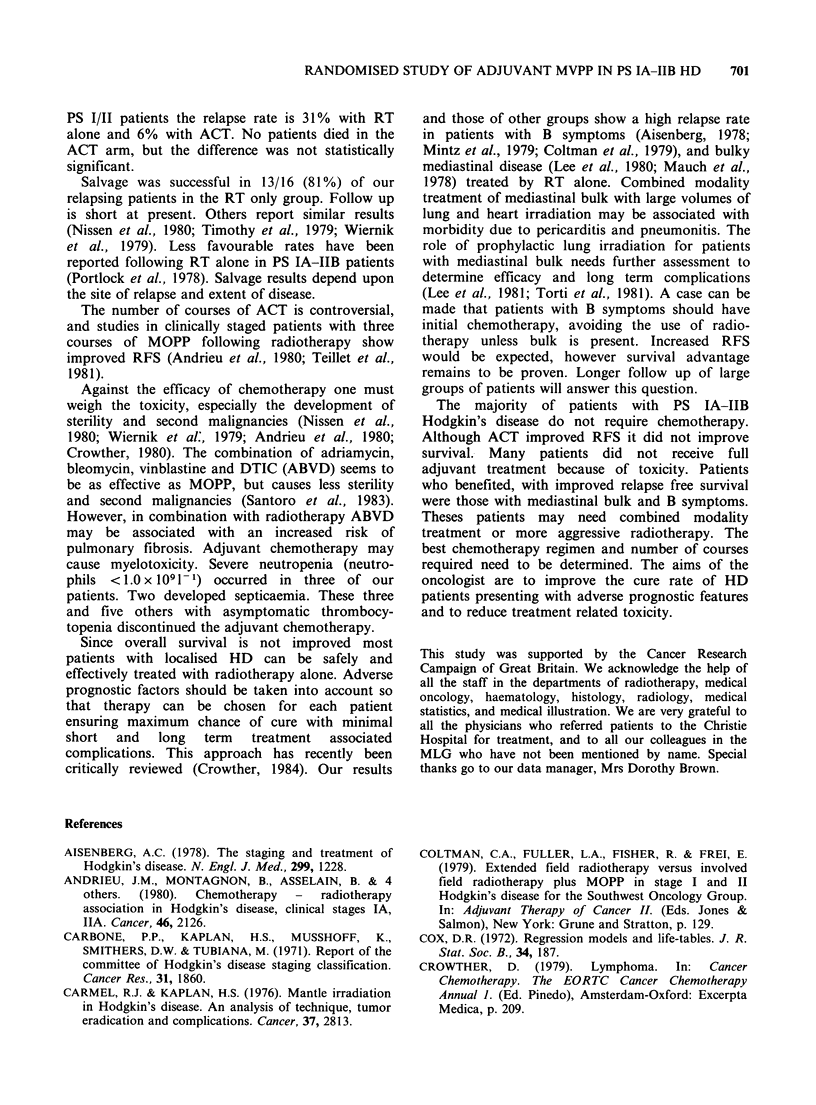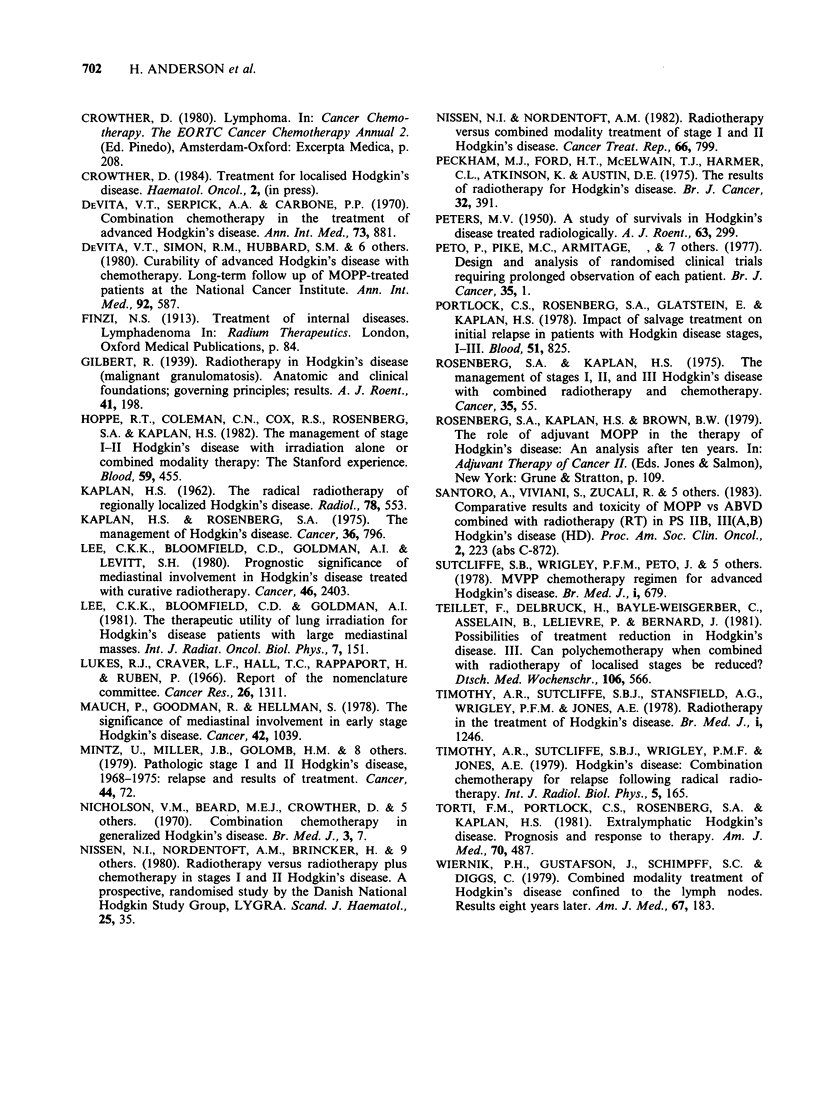# A randomised study of adjuvant chemotherapy after mantle radiotherapy in supradiaphragmatic Hodgkin's disease PS IA-IIB: a report from the Manchester lymphoma group.

**DOI:** 10.1038/bjc.1984.110

**Published:** 1984-06

**Authors:** H. Anderson, D. P. Deakin, J. Wagstaff, J. M. Jones, I. D. Todd, P. M. Wilkinson, R. D. James, W. P. Steward, G. Blackledge, J. H. Scarffe

## Abstract

One hundred and fourteen untreated patients with pathological stage (PS) IA-IIB supradiaphragmatic Hodgkin's Disease were randomised to mantle radiotherapy alone (55) or mantle radiotherapy followed by 6 courses of adjuvant chemotherapy with mustine, vinblastine, prednisolone and procarbazine- MVPP (59). Patients excluded were those outside the age range 16-65 years and those with massive mediastinal disease precluding laparotomy. Bulk disease was defined as a mass of lymph nodes measuring five centimetres or more in any axis. Mediastinal bulk was present if the ratio of the maximum width of mediastinal disease to the maximal chest diameter was more than one third. All patients achieved a complete remission. Median duration of follow-up was 62 months (range 16-97). The relapse free survival (RFS) was 81%; 69% for radiotherapy alone and 93% for adjuvant chemotherapy (P = 0.002). RFS was also shown to be adversely affected by B symptoms (P = 0.0003), bulk disease (P = 0.018), abnormal CXR (P = 0.037), and increasing stage (P = 0.039). Age, sex, histology, and number of sites involved had no significant effect upon RFS. A Cox multivariate analysis showed that only three variables had a significant adverse effect on RFS - radiotherapy alone, the presence of bulk disease, and B symptoms. The overall 5 year survival was 93% with no statistically significant difference between the two treatment groups (P = 0.54). Survival was adversely affected by three variables - B symptoms (P = 0.02), the presence of bulk disease (P = 0.002), and pathological stage (P = 0.05). High risk groups for relapse are those with bulk and B symptoms. This analysis has shown that RFS was significantly improved by adjuvant chemotherapy, but that overall survival was not.


*Br. J. of Cancer* (1984), **49**, 695–702

### A randomised study of adjuvant chemotherapy after mantle radiotherapy in supradiaphragmatic Hodgkin's disease PS IA-IIB: A report from the Manchester lymphoma group

H. Anderson[1], D.P. Deakin[2], J. Wagstaff[1], J.M. Jones[3], I.D.H. Todd[2], P.M. Wilkinson[2], R.D. James[2], W.P. Steward[1], G. Blackledge[4], J.H. Scarffe[1] & D. Crowther[1]

[1]*Dept. Medical Oncology,* [2]*Dept. Radiotherapy, and* [3]*Dept. Medical Statistics, Christie Hospital, Manchester, M20 9BX,* [4]*Dept. Clinical Oncology, Queen Elizabeth Hospital, Birmingham 15, UK.*

**Summary** One hundred and fourteen untreated patients with pathological stage (PS) IA-IIB supradiaphragmatic Hodgkin's Disease were randomised to mantle radiotherapy alone (55) or mantle radiotherapy followed by 6 courses of adjuvant chemotherapy with mustine, vinblastine, prednisolone and procarbazine- MVPP (59). Patients excluded were those outside the age range 16–65 years and those with massive mediastinal disease precluding laparotomy. Bulk disease was defined as a mass of lymph nodes measuring five centimetres or more in any axis. Mediastinal bulk was present if the ratio of the maximum width of mediastinal disease to the maximal chest diameter was more than one third..

All patients achieved a complete remission. Median duration of follow-up was 62 months (range 16–97). The relapse free survival (RFS) was 81%; 69% for radiotherapy alone and 93% for adjuvant chemotherapy ($P=0.002$). RFS was also shown to be adversely affected by B symptoms ($P=0.0003$), bulk disease ($P=0.018$), abnormal CXR ($P=0.037$), and increasing stage ($P=0.039$). Age, sex, histology, and number of sites involved had no significant effect upon RFS. A Cox multivariate analysis showed that only three variables had a significant adverse effect on RFS – radiotherapy alone, the presence of bulk disease, and B symptoms. The overall 5 year survival was 93% with no statistically significant difference between the two treatment groups ($P=0.54$). Survival was adversely affected by three variables – B symptoms ($P=0.02$), the presence of bulk disease ($P=0.002$), and pathological stage ($P=0.05$).

High risk groups for relapse are those with bulk and B symptoms. This analysis has shown that RFS was significantly improved by adjuvant chemotherapy, but that overall survival was not.

In 1913 Finzi advocated irradiation of uninvolved sites in patients with Hodgkin's disease in an attempt to procure long term RFS (Finzi, 1913). The work of Gilbert (1939), Peters (1950), and Kaplan (1962), led to the general acceptance of megavoltage extended field radiotherapy for patients with localised Hodgkin's disease because of the greatly improved RFS. Relapses occur following radiotherapy alone, and relapse rates of 20–30% have been associated with mantle radiotherapy in stage IA–IIB patients (Carmel & Kaplan, 1976; Peckham *et al.*, 1975; Timothy *et al.*, 1978). Kaplan defined adverse prognostic factors for his patients and then advocated radiotherapy ranging from involved field treatment for favourable histology (pathologically staged IA disease in favourable sites), to total nodal irradiation for PS IB/IIB disease (Kaplan & Rosenberg, 1975). However, even when extended field therapy with lung irradiation to patients with a high risk of relapse was given the overall supradiaphragmatic relapse rate was 20% in PS I and II disease (Carmel & Kaplan, 1976).

Combination chemotherapy developed by DeVita *et al.* (1970) improved the cure rate of patients with advanced stage HD. MOPP or MVPP (Sutcliffe *et al.*, 1978), are standard treatments for stage IIIB/IV disease. When chemotherapy is used to salvage relapsed patients whose localised disease was treated by radiotherapy alone only about half are found to have long term survival (Portlock *et al.*, 1978). For these reasons we initiated a randomised trial in 1974 to assess the role of adjuvant MVPP in improving RFS and overall survival in PS IA–IIB disease. It was also hoped that adverse prognostic factors at presentation would allow identification of patients who might benefit from adjuvant chemotherapy (ACT).

The trial protocol was written in 1974 when there were few adjuvant trials in progress. Now several reports of adjuvant therapy are available for PS IA–IIB Hodgkin's disease. (Coltman *et al.*, 1979; Wiernik *et al.*, 1979; Hoppe *et al.*, 1982; Nissen & Nordentoft, 1982).

Correspondence: H. Anderson
Received 12 December 1983; accepted 27 February 1984.

© The Macmillan Press Ltd., 1984

This paper describes the results of a randomised controlled trial of adjuvant MVPP in 115 patients after median follow-up of 62 months.

### Patients and methods

From October 1974 to August 1981 patients with newly diagnosed pathologically staged supradiaphragmatic Hodgkin's disease (HD) were treated by mantle radiotherapy and then randomised to receive ACT with six courses of mustine, vinblastine, prednisolone and procarbazine (MVPP) or follow-up. Patients were excluded from the trial if massive mediastinal disease prevented laparotomy staging by making the anaesthetic risk too high, or were outside the age range 16–65 years. One hundred and fifteen patients were entered into the trial, but one has been excluded from analysis because he was lost from follow up.

Patients had routine haematological and biochemical tests. In addition lymphangiograms were performed on patients prior to 1976. From 1976 abdominal computerised tomograms were performed to aid staging. All patients had a routine staging laparotomy and bone marrow biopsy. The pathology was reviewed in all cases and classified according to the Rye classification (Lukes *et al.*, 1966). Staging was according to the Ann Arbor classification (Carbone *et al.*, 1971).

Bulk disease was defined as a mass of lymph nodes outside the chest measuring $\geq 5$ cm in any one axis. Mediastinal bulk was present if the ratio of the maximum width of mediastinal disease to the maximum chest diameter was one third or more. Patients were examined at first review after radiotherapy for remission status. A complete remission was defined as absence of all clinical, biochemical and radiological evidence of disease. Patients were then randomised to ACT or follow up alone.

### Treatment

The radiotherapy was given by the "mantle" technique. Anterior and posterior opposed 4 Mv fields were used to encompass the major node areas in the upper half of the body. The fields extended from the external auditory meatus superiorly to the eleventh thoracic vertebra inferiorly, covering the axillae laterally. The dose delivered at the mid-plane was 3500 cGy in 20 fractions, in 4 weeks. From the onset of treatment the majority of lung tissue was shielded by individually fashioned blocks. The spinal cord was shielded for half the posterior treatments, and a compensatory anterior mediastinal lung field was used to deliver 600 cGy on the final treatment day. There was consequently a dose gradient from 2900 cGy in the posterior mediastinum to 3900 cGy in the anterior mediastinum.

The adjuvant chemotherapy given was 6 courses of MVPP at 6-weekly intervals, mustine 6 mg m$^{-2}$ (max. 10 mg) IV days 1 & 8, vinblastine 6 mg m$^{-2}$ (max 10 mg) IV days 1 & 8, prednisolone 40 mg day$^{-1}$ orally days 1–14, and procarbazine 100 mg m$^{-2}$ orally days 1–14.

### *Patient review*

Patients have been reviewed regularly in the clinic. (After treatment had finished they were reviewed monthly for the first year, 2-monthly in the second, 3-monthly in the third, four monthly in the fourth, 6-monthly in the fifth, and yearly thereafter). The median duration of follow up was 62 months (range 16–97 months).

### *Statistical methods*

The effects of several variables on survival and RFS have been analysed by calculating Kaplan–Meier curves, which were then compared using the logrank test (Peto *et al.*, 1977). Cox's proportional hazards model (Cox, 1972) had been used to determine the most significant variables tnat affect RFS. Both overall survival and RFS have been calculated from the completion of radiotherapy.

### Results

Fifty-five patients were in the radiotherapy (RT) alone group, and 59 in the ACT group. Patient characteristics were similar in the two groups (Table I). All patients attained a complete

**Table I** Patient characteristics of each treatment group.

| | *RT alone* | *RT+MVPP* | *Total* |
|---|---|---|---|
| No. patients | 55 | 59 | 114 |
| Male | 38 | 39 | 77 |
| Female | 17 | 20 | 37 |
| Stage IA | 24 | 23 | 47 |
| IB | 2 | 2 | 4 |
| IIA | 19 | 25 | 44 |
| IIB | 10 | 9 | 19 |
| Age years (mean) | 32.5 | 31.5 | |
| CXR normal | 31 | 35 | 66 |
| abnormal | 24 | 24 | 48 |
| Bulk absent | 37 | 34 | 71 |
| present | 18 | 25 | 43 |
| Histology LP | 16 | 20 | 36 |
| NS | 22 | 24 | 46 |
| MC | 17 | 15 | 32 |

LP = Lymphocyte predominant, NS = nodular sclerosing, MC = Mixed Cellularity.

remission. This occurred after the start of chemotherapy in two patients. Five patients had an extra radiotherapy field to areas of remaining bulk disease after mantle treatment, four to the neck and one to the infraclavicular area. Two of these 5 patients were in the RT group.

Twenty-five of the patients randomised to ACT received less than 6 courses of MVPP. Seven received no chemotherapy at all – two patients refused chemotherapy after randomisation, 3 patients were not given MVPP on medical grounds (one had severe post RT lethargy and weight loss, one had hepatitis on liver biopsy, and one had an isolated C7 nerve palsy investigated until it was too late to give MVPP), and two had no MVPP for technical reasons (difficulties with venepuncture). These 7 patients have been included in the ACT group for statistical analysis in order to avoid any bias in the treatment comparisons.

Of the other 18 patients, 8 discontinued chemotherapy after developing myelosuppression (two developed septicaemia), 5 after developing mild infections, two refused further chemotherapy due to toxicity, 3 were stopped due to toxicity (one had severe gastrointestinal colic, one had steroid induced depression and suicidal tendency, and one had severe lethargy).

*Relapse free survival (RFS)*

The overall 5 year RFS was 81%; 69% in the RT group and 93% in the ACT group ($P=0.002$). (Figure 1). Twenty patients have relapsed, 16/55 in the RT group (29%) and 4/59 in the ACT group (7%). In the latter group two who had a CR with radiotherapy alone, relapsed through ACT and died, and one did not receive adjuvant treatment following the finding of hepatitis at laparotomy liver biopsy (discussed later). The fourth patient received full ACT. Nine variables were analysed for effect upon RFS and survival – age, sex, histology, pathological stage, A/B symptoms, number of sites involved, CXR status, bulk, and treatment. The results are shown in Table II. The presence of B symptoms was the most significant variable ($P=0.0003$), adversely affecting RFS. RT alone was associated with a higher probability of relapse than RT+ ACT ($P=0.002$). Bulky disease was associated with poor RFS ($P=0.018$), as was an abnormal CXR ($P=0.037$), and increasing stage ($P=0.039$). The other variables had no statistically significant effect upon RFS (number of sites involved, histology, age, and sex). There was no statistically significant difference in RFS between those receiving full course ACT and those who received less chemotherapy than planned.

A Cox's multivariate analysis was performed to determine those variables with the most significant effect upon RFS. The results showed that the most significant variable was treatment group, patients receiving ACT having fewer relapses. This was followed in significance by bulk then by B symptoms, both adversely affecting RFS (Table III). The other variables were no longer significant. With these three variables in the model the others do not add significantly to the ability to predict RFS. Variables previously significant in the logrank test (stage and CXR status), were both correlated with bulk and B symptoms. ACT significantly

**Figure 1** Relapse free survival for all 114 patients with PS IA–IIB Hodgkin's disease. Relationship to treatment given.

**Table II** Variables analysed for effect upon survival and RFS.

| Variable | *P value Logrank test* Survival | RFS |
|---|---|---|
| Age<36≥36 | 0.67 | 0.45 |
| Sex | 0.76 | 0.79 |
| Histology (LP, NS, MC) | 0.30 | 0.18 |
| Pathological stage | 0.05 | 0.039[a] |
| A/B symptoms | 0.02[a] | 0.0003[a] |
| No. sites involved (1,2,3,4+) | 0.22 | 0.10 |
| CXR normal/abnormal | 0.10 | 0.037[a] |
| Bulky disease absent/present | 0.002[a] | 0.018[a] |
| Treatment group | 0.54 | 0.002[a] |

[a]*P* value statistically significant.

**Table III** Cox's multivariate analysis of factors affecting RFS.

| | *P value* | *Favourable feature* |
|---|---|---|
| Treatment | 0.001 | MVPP |
| Bulk | 0.009 | Absent |
| B symptoms | 0.029 | Absent |

**Figure 2** Relapse free survival for the subgroup of 54 patients with adverse prognostic features at presentation i.e. bulk disease and/or B symptoms. Relationship to treatment given.

**Table IV** Relapse rate according to pathological stage and treatment given.

| *Stage* | *RT only (55)*[a] | *RT+MVPP (59)*[a] | *Overall (114)*[a] |
|---|---|---|---|
| 1A | 3/24 (12.5%) | 1/23 (4%) | 4/47 (8.5%) |
| 1B | 1/2 | 0/2 | 1/4 |
| IIA | 6/19 (32%) | 1/25 (4%) | 7/44 (16%) |
| IIB | 6/10 (60%) | 2/9 (22%) | 8/19 (42%) |
| Total | 16/55 (29%) | 4/59 (7%) | 20/114 (17.5%) |

[a]No. patients.

**Table V** Relapse rate associated with the presence or absence of bulk disease.

| | *RT only no. relapsing/total* | *RT+MVPP no. relapsing/total* |
|---|---|---|
| Mediast bulk | 3/4 (75%) | 1/5 (20%) |
| Mediast bulk + extrathoracic bulk | 2/4 (50%) | 1/5 (20%) |
| Extrathoracic bulk only | 4/10 (40%) | 2/15 (13%) |
| No bulk | 7/37 (19%) | 0/34 (0%) |
| Total | 16/55 (29%) | 4/59 (7%) |

improved RFS in the 54 patients with B symptoms or bulk disease (Figure 2).

*Relapses*

Analysis of relapse by stage and treatment group is shown in Table IV. There were 4 relapses in the ACT group, and 16 in the RT only group. Overall 4/47 (8.5%) stage IA, 7/44 (16%) stage IIA, 1/4 (25%) stage IB, 8/19 (42%) stage IIB patients relapsed. Analysing stage IIB alone the relapse rate was 6/10 (60%) in the RT group, and 2/9 (22%) in the ACT group.

Twelve of the 20 patients who relapsed had bulk disease at presentation, 8/16 in the RT group, and 4/4 in the ACT group. Analysis of relapse by bulk and treatment group is shown in Table V. If bulk disease was present relapse involving the primary site occurred in 6/12 cases, whereas none of the relapses occurring in patients without bulky disease occurred in the site of initial disease. Mediastinal bulk was present in 8/55 patients treated by RT alone, and 10/59 treated with ACT. Relapse occurred in seven of these 18 patients (39%), 5/8 RT (62%), and 2/10 ACT (20%).

An analysis of the site of relapse in relation to radiotherapy field showed nine relapses outside the field, six within, and five relapses occurred simultaneously inside and outside the radiotherapy field. Ten relapses occurred in nodes alone, and 6 in extranodal sites alone (one in bone, 2 in liver and bone, one in the lung, liver, diaphragm, and pericardium, one in the pleura and chest wall, and one in the humerus and overlying skin). Four patients had simultaneous nodal and extranodal relapse (3 mediastinum and lung, and one in para-aortic glands and bone).

*Salvage treatment*

Of the 4 patients relapsing in the ACT group, 2 relapsed during ACT and died of HD (both had PS IIB disease with mediastinal bulk at presentation). One patient with pulmonary and hilar relapse failed to respond to MVPP or radiotherapy and died of disease. The fourth patient received no ACT (hepatitis on liver biopsy), and achieved a CR on MVPP.

Sixteen relapsed after RT alone, and 13 achieved a CR on chemotherapy (11 MVPP and 2 Chlorambucil VPP). Seven of these patients received adjuvant radiotherapy to sites of relapse. One patient received no treatment because the relapse was diagnosed at *post mortem*. Another patient failed to achieve a CR after relapse in the skin and bone and died of HD, septicaemia and disseminated herpes zoster. The remaining patient achieved a CR using MVPP after mediastinal and pleural relapse. He then had a second relapse and died of disseminated herpes zoster and HD.

*Survival*

The overall 5 year survival rate was 93%. There was no statistically significant difference between the two treatment groups (94% for RT alone and 91% for ACT $P=0.54$ – Figure 3). The logrank test was used to determine the effect of nine variables on survival. The results are shown in Table II. Survival was adversely affected by the presence of bulk disease ($P=0.002$), and B symptoms ($P=0.02$). Pathological stage was of borderline significance ($P=0.05$) – stage II patients doing less well than stage I. The other variables were of no prognostic significance. A multivariate analysis of these variables was not possible, owing to the small number of deaths involved. Figure 4 shows no significant survival advantage to patients receiving ACT when bulk and/or B symptoms were present.

**Figure 3** Overall survival for all 114 patients with PS IA–IIB Hodgkin's disease. Relationship to treatment given.

**Figure 4** Overall survival for the subgroup of 54 patients with adverse prognostic features at presentation i.e. bulk disease and/or B symptoms. Relationship to treatment given.

*Deaths*

There were 8 deaths, 3 in the radiotherapy alone group and 5 in the ACT group. (PS IA (1), IIA (3), IIB (4)). They all had bulky disease at presentation.

Two patients died from intercurrent causes – one following dental extraction, and the other from a secondary carcinoma of unknown primary site. The first patient died in complete remission, two months after finishing MVPP chemotherapy for relapsed HD. He was in the ACT group (but received no chemotherapy because the laparotomy liver biopsy showed changes of hepatitis.) He died of pneumococcal septicaemia 5 days after dental treatment. The other patient had only one course of adjuvant treatment because she developed septicaemia when leucopenic. Her second malignancy became apparent 4 months after attaining a complete remission from Hodgkin's disease.

Of the remaining 6 patients dying with active disease, 3 were in each treatment group. Two receiving ACT relapsed through chemotherapy. The other relapsed with mediastinal and lung disease and died of pneumonia and progressing HD. Of the three dying after radiotherapy alone, in one patient HD was identified in the paratracheal glands at postmortem examination following death from *E.coli* septicaemia. There had been no clinical evidence of relapse. Of the other two, one died in his first, and the other in his second relapse.

### Discussion

This trial of adjuvant chemotherapy following radiotherapy for localised Hodgkin's disease was different from other randomised trials in two respects – the radiotherapy was the same in both arms of the trial (mantle RT), and MVPP was used rather than MOPP. It has been shown that remission rates and survival in advanced Hodgkin's disease are similar for MOPP and MVPP therapy, however MVPP has less neurotoxicity (Nicholson *et al.*, 1970; Sutcliffe *et al.*, 1978; Crowther, 1979; DeVita *et al.*, 1980).

We have shown that 5 year RFS for PS IA–IIB HD patients has been significantly improved by adjuvant MVPP, although there was no improvement in overall survival. Four other randomised studies of adjuvant chemotherapy in patients with localised pathologically staged HD have been published. They are summarised in Table VI. Stanford compared radiotherapy alone using varied fields (involved field, mantle, ±lung or sub-diaphragmatic irradiation depending on the patient prognostic factors), with involved or extended field radiotherapy followed by 6 courses of MOPP (Rosenberg & Kaplan, 1975; Rosenberg *et al.*, 1979; Hoppe *et al.*, 1982). In their study, ACT significantly improved RFS in PS IA/IIA disease but not in IB/IIB disease. This contrasts with our study which showed benefit for patients with B symptoms following ACT. The probable reason for this difference may be the more extensive radiotherapy fields given at Stanford to patients with B symptoms in the RT alone arm. Stanford showed borderline survival advantage at 10 years follow-up after ACT to patients with IA/IIA disease but the overall survival of Stanford patients with mediastinal bulk at presentation was the same in both arms of the trial.

The Southwest Oncology Group (SWOG) has also shown an improved RFS after ACT. The compared involved field RT plus adjuvant MOPP with extended field RT alone, and they found mediastinal disease, E disease and B symptoms to be adverse prognostic factors associated with increased RFS when given ACT. They also failed to show an overall survival advantage from ACT (Coltman *et al.*, 1979).

A study from Denmark compared TNI with mantle RT followed by six courses of MOPP. This again showed RFS benefit from ACT but no overall survival advantage (Nissen & Nordentoft, 1982).

Wiernik *et al.* (1979) randomised 87 PS IA, IIA/B and IIIA patients to receive extended field radiotherapy followed by adjuvant MOPP or follow up. Their median follow up is 69 months. For 46

**Table VI** Summary of randomised trials of adjuvant combination chemotherapy in PSIA-IIB Hodgkin's disease.

| *Authors* | *No.* | *Years* | *Treatment* | *Adjuvant chemotherapy significantly improves* *RFS* | *Survival* |
|---|---|---|---|---|---|
| Coltman *et al.*, 1979 | 206 | 1972–1978 | EF<br>IF+6MOPP | Yes | No |
| Hoppe *et al.*, 1982 | 230 | 1968–1978 | EF/STLI/TLI<br>IF/STNI/TNI+6MOPP | Yes-only med.bulk | No |
| Nissen *et al.*, 1982 | 261 | 1971–1980 | TNI<br>Mantle+6MOPP | Yes | No |
| Wiernik *et al.*, 1979 | 46 | 1970–1974 | EF<br>EF+6MOPP | Yes | No |

IF = Involved field radiotherapy.
EF = Extended field radiotherapy.
STNI = subtotal nodal irradiation.
TNI = total nodal irradiation.

PS I/II patients the relapse rate is 31% with RT alone and 6% with ACT. No patients died in the ACT arm, but the difference was not statistically significant.

Salvage was successful in 13/16 (81%) of our relapsing patients in the RT only group. Follow up is short at present. Others report similar results (Nissen *et al.*, 1980; Timothy *et al.*, 1979; Wiernik *et al.*, 1979). Less favourable rates have been reported following RT alone in PS IA–IIB patients (Portlock *et al.*, 1978). Salvage results depend upon the site of relapse and extent of disease.

The number of courses of ACT is controversial, and studies in clinically staged patients with three courses of MOPP following radiotherapy show improved RFS (Andrieu *et al.*, 1980; Teillet *et al.*, 1981).

Against the efficacy of chemotherapy one must weigh the toxicity, especially the development of sterility and second malignancies (Nissen *et al.*, 1980; Wiernik *et al.*, 1979; Andrieu *et al.*, 1980; Crowther, 1980). The combination of adriamycin, bleomycin, vinblastine and DTIC (ABVD) seems to be as effective as MOPP, but causes less sterility and second malignancies (Santoro *et al.*, 1983). However, in combination with radiotherapy ABVD may be associated with an increased risk of pulmonary fibrosis. Adjuvant chemotherapy may cause myelotoxicity. Severe neutropenia (neutrophils $<1.0 \times 10^9 l^{-1}$) occurred in three of our patients. Two developed septicaemia. These three and five others with asymptomatic thrombocytopenia discontinued the adjuvant chemotherapy.

Since overall survival is not improved most patients with localised HD can be safely and effectively treated with radiotherapy alone. Adverse prognostic factors should be taken into account so that therapy can be chosen for each patient ensuring maximum chance of cure with minimal short and long term treatment associated complications. This approach has recently been critically reviewed (Crowther, 1984). Our results and those of other groups show a high relapse rate in patients with B symptoms (Aisenberg, 1978; Mintz *et al.*, 1979; Coltman *et al.*, 1979), and bulky mediastinal disease (Lee *et al.*, 1980; Mauch *et al.*, 1978) treated by RT alone. Combined modality treatment of mediastinal bulk with large volumes of lung and heart irradiation may be associated with morbidity due to pericarditis and pneumonitis. The role of prophylactic lung irradiation for patients with mediastinal bulk needs further assessment to determine efficacy and long term complications (Lee *et al.*, 1981; Torti *et al.*, 1981). A case can be made that patients with B symptoms should have initial chemotherapy, avoiding the use of radiotherapy unless bulk is present. Increased RFS would be expected, however survival advantage remains to be proven. Longer follow up of large groups of patients will answer this question.

The majority of patients with PS IA–IIB Hodgkin's disease do not require chemotherapy. Although ACT improved RFS it did not improve survival. Many patients did not receive full adjuvant treatment because of toxicity. Patients who benefited, with improved relapse free survival were those with mediastinal bulk and B symptoms. Theses patients may need combined modality treatment or more aggressive radiotherapy. The best chemotherapy regimen and number of courses required need to be determined. The aims of the oncologist are to improve the cure rate of HD patients presenting with adverse prognostic features and to reduce treatment related toxicity.

This study was supported by the Cancer Research Campaign of Great Britain. We acknowledge the help of all the staff in the departments of radiotherapy, medical oncology, haematology, histology, radiology, medical statistics, and medical illustration. We are very grateful to all the physicians who referred patients to the Christie Hospital for treatment, and to all our colleagues in the MLG who have not been mentioned by name. Special thanks go to our data manager, Mrs Dorothy Brown.

### References

AISENBERG, A.C. (1978). The staging and treatment of Hodgkin's disease. *N. Engl. J. Med.*, **299**, 1228.

ANDRIEU, J.M., MONTAGNON, B., ASSELAIN, B. & 4 others. (1980). Chemotherapy – radiotherapy association in Hodgkin's disease, clinical stages IA, IIA. *Cancer*, **46**, 2126.

CARBONE, P.P., KAPLAN, H.S., MUSSHOFF, K., SMITHERS, D.W. & TUBIANA, M. (1971). Report of the committee of Hodgkin's disease staging classification. *Cancer Res.*, **31**, 1860.

CARMEL, R.J. & KAPLAN, H.S. (1976). Mantle irradiation in Hodgkin's disease. An analysis of technique, tumor eradication and complications. *Cancer*, **37**, 2813.

COLTMAN, C.A., FULLER, L.A., FISHER, R. & FREI, E. (1979). Extended field radiotherapy versus involved field radiotherapy plus MOPP in stage I and II Hodgkin's disease for the Southwest Oncology Group. In: *Adjuvant Therapy of Cancer II.* (Eds. Jones & Salmon), New York: Grune and Stratton, p. 129.

COX, D.R. (1972). Regression models and life-tables. *J. R. Stat. Soc. B.*, **34**, 187.

CROWTHER, D. (1979). Lymphoma. In: *Cancer Chemotherapy. The EORTC Cancer Chemotherapy Annual 1.* (Ed. Pinedo), Amsterdam-Oxford: Excerpta Medica, p. 209.

CROWTHER, D. (1980). Lymphoma. In: *Cancer Chemotherapy. The EORTC Cancer Chemotherapy Annual 2.* (Ed. Pinedo), Amsterdam-Oxford: Excerpta Medica, p. 208.

CROWTHER, D. (1984). Treatment for localised Hodgkin's disease. *Haematol. Oncol.*, **2**, (in press).

DEVITA, V.T., SERPICK, A.A. & CARBONE, P.P. (1970). Combination chemotherapy in the treatment of advanced Hodgkin's disease. *Ann. Int. Med.*, **73**, 881.

DEVITA, V.T., SIMON, R.M., HUBBARD, S.M. & 6 others. (1980). Curability of advanced Hodgkin's disease with chemotherapy. Long-term follow up of MOPP-treated patients at the National Cancer Institute. *Ann. Int. Med.*, **92**, 587.

FINZI, N.S. (1913). Treatment of internal diseases. Lymphadenoma In: *Radium Therapeutics*. London, Oxford Medical Publications, p. 84.

GILBERT, R. (1939). Radiotherapy in Hodgkin's disease (malignant granulomatosis). Anatomic and clinical foundations; governing principles; results. *A. J. Roent.*, **41**, 198.

HOPPE, R.T., COLEMAN, C.N., COX, R.S., ROSENBERG, S.A. & KAPLAN, H.S. (1982). The management of stage I–II Hodgkin's disease with irradiation alone or combined modality therapy: The Stanford experience. *Blood*, **59**, 455.

KAPLAN, H.S. (1962). The radical radiotherapy of regionally localized Hodgkin's disease. *Radiol.*, **78**, 553.

KAPLAN, H.S. & ROSENBERG, S.A. (1975). The management of Hodgkin's disease. *Cancer*, **36**, 796.

LEE, C.K.K., BLOOMFIELD, C.D., GOLDMAN, A.I. & LEVITT, S.H. (1980). Prognostic significance of mediastinal involvement in Hodgkin's disease treated with curative radiotherapy. *Cancer*, **46**, 2403.

LEE, C.K.K., BLOOMFIELD, C.D. & GOLDMAN, A.I. (1981). The therapeutic utility of lung irradiation for Hodgkin's disease patients with large mediastinal masses. *Int. J. Radiat. Oncol. Biol. Phys.*, **7**, 151.

LUKES, R.J., CRAVER, L.F., HALL, T.C., RAPPAPORT, H. & RUBEN, P. (1966). Report of the nomenclature committee. *Cancer Res.*, **26**, 1311.

MAUCH, P., GOODMAN, R. & HELLMAN, S. (1978). The significance of mediastinal involvement in early stage Hodgkin's disease. *Cancer*, **42**, 1039.

MINTZ, U., MILLER, J.B., GOLOMB, H.M. & 8 others. (1979). Pathologic stage I and II Hodgkin's disease, 1968–1975: relapse and results of treatment. *Cancer*, **44**, 72.

NICHOLSON, V.M., BEARD, M.E.J., CROWTHER, D. & 5 others. (1970). Combination chemotherapy in generalized Hodgkin's disease. *Br. Med. J.*, **3**, 7.

NISSEN, N.I., NORDENTOFT, A.M., BRINCKER, H. & 9 others. (1980). Radiotherapy versus radiotherapy plus chemotherapy in stages I and II Hodgkin's disease. A prospective, randomised study by the Danish National Hodgkin Study Group, LYGRA. *Scand. J. Haematol.*, **25**, 35.

NISSEN, N.I. & NORDENTOFT, A.M. (1982). Radiotherapy versus combined modality treatment of stage I and II Hodgkin's disease. *Cancer Treat. Rep.*, **66**, 799.

PECKHAM, M.J., FORD, H.T., MCELWAIN, T.J., HARMER, C.L., ATKINSON, K. & AUSTIN, D.E. (1975). The results of radiotherapy for Hodgkin's disease. *Br. J. Cancer*, **32**, 391.

PETERS, M.V. (1950). A study of survivals in Hodgkin's disease treated radiologically. *A. J. Roent.*, **63**, 299.

PETO, P., PIKE, M.C., ARMITAGE, , & 7 others. (1977). Design and analysis of randomised clinical trials requiring prolonged observation of each patient. *Br. J. Cancer*, **35**, 1.

PORTLOCK, C.S., ROSENBERG, S.A., GLATSTEIN, E. & KAPLAN, H.S. (1978). Impact of salvage treatment on initial relapse in patients with Hodgkin disease stages, I–III. *Blood*, **51**, 825.

ROSENBERG, S.A. & KAPLAN, H.S. (1975). The management of stages I, II, and III Hodgkin's disease with combined radiotherapy and chemotherapy. *Cancer*, **35**, 55.

ROSENBERG, S.A., KAPLAN, H.S. & BROWN, B.W. (1979). The role of adjuvant MOPP in the therapy of Hodgkin's disease: An analysis after ten years. In: *Adjuvant Therapy of Cancer II.* (Eds. Jones & Salmon), New York: Grune & Stratton, p. 109.

SANTORO, A., VIVIANI, S., ZUCALI, R. & 5 others. (1983). Comparative results and toxicity of MOPP vs ABVD combined with radiotherapy (RT) in PS IIB, III(A,B) Hodgkin's disease (HD). *Proc. Am. Soc. Clin. Oncol.*, **2**, 223 (abs C-872).

SUTCLIFFE, S.B., WRIGLEY, P.F.M., PETO, J. & 5 others. (1978). MVPP chemotherapy regimen for advanced Hodgkin's disease. *Br. Med. J.*, **i**, 679.

TEILLET, F., DELBRUCK, H., BAYLE-WEISGERBER, C., ASSELAIN, B., LELIEVRE, P. & BERNARD, J. (1981). Possibilities of treatment reduction in Hodgkin's disease. III. Can polychemotherapy when combined with radiotherapy of localised stages be reduced? *Dtsch. Med. Wochenschr.*, **106**, 566.

TIMOTHY, A.R., SUTCLIFFE, S.B.J., STANSFIELD, A.G., WRIGLEY, P.F.M. & JONES, A.E. (1978). Radiotherapy in the treatment of Hodgkin's disease. *Br. Med. J.*, **i**, 1246.

TIMOTHY, A.R., SUTCLIFFE, S.B.J., WRIGLEY, P.M.F. & JONES, A.E. (1979). Hodgkin's disease: Combination chemotherapy for relapse following radical radiotherapy. *Int. J. Radiol. Biol. Phys.*, **5**, 165.

TORTI, F.M., PORTLOCK, C.S., ROSENBERG, S.A. & KAPLAN, H.S. (1981). Extralymphatic Hodgkin's disease. Prognosis and response to therapy. *Am. J. Med.*, **70**, 487.

WIERNIK, P.H., GUSTAFSON, J., SCHIMPFF, S.C. & DIGGS, C. (1979). Combined modality treatment of Hodgkin's disease confined to the lymph nodes. Results eight years later. *Am. J. Med.*, **67**, 183.